# Electrostatics-Based Computational Design and Experimental Analysis of Buforin II Antimicrobial Peptide Variants with Increased DNA Affinities

**DOI:** 10.1021/acsomega.3c04023

**Published:** 2023-09-01

**Authors:** Qiao Li, Gabriela Kim, Lisha Jing, Xiaoxuan Ji, Donald E. Elmore, Mala L. Radhakrishnan

**Affiliations:** † Biochemistry Program, Wellesley College, Wellesley, Massachusetts 02481, United States; ‡ Chemistry Department, Wellesley College, Wellesley, Massachusetts 02481, United States

## Abstract

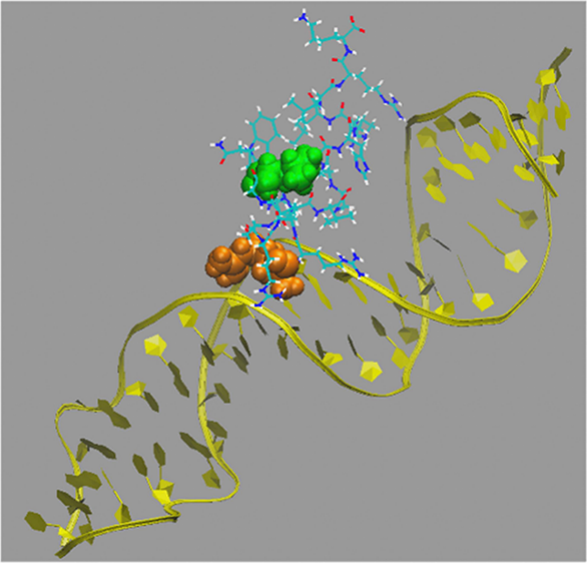

Antimicrobial peptides (AMPs) are promising alternatives to traditional antibiotics in the treatment of bacterial infections in part due to their targeting of generic bacterial structures that make it more difficult to develop drug resistance. In this study, we introduce and implement a design workflow to develop more potent AMPs by improving their electrostatic interactions with DNA, which is a putative intracellular target. Using the existing membrane-translocating AMP buforin II (BF2) as a starting point, we use a computational workflow that integrates electrostatic charge optimization, continuum electrostatics, and molecular dynamics simulations to suggest peptide positions at which a neutral BF2 residue could be substituted with arginine to increase DNA-binding affinity either significantly or minimally, with the latter choice done to determine whether AMP binding affinity depends on charge distribution and not just overall monopole. Our analyses predicted that T1R and L8R BF2 variants would yield substantial and minimal increases in DNA-binding affinity, respectively. These predictions were validated with experimental peptide-DNA binding assays with additional computational analyses providing structural insights. Additionally, experimental measurements of antimicrobial potency showed that a design to increase DNA binding can also yield greater potency. As a whole, this study takes initial steps to support the idea that (i) a design strategy aimed to increase AMP binding affinity to DNA by focusing only on electrostatic interactions can improve AMP potency and (ii) the effect on DNA binding of increasing the overall peptide monopole via arginine substitution depends on the position of the substitution. More broadly, this design strategy is a novel way to increase the potency of other membrane-translocating AMPs that target nucleic acids.

## Introduction

Although the development of new antibiotics has dramatically slowed in recent decades, there is an acute need for developing new and improved therapeutic approaches for treating bacterial infections.^1,2^ Recognizing that need, many researchers have noted the potential promise of antimicrobial peptides (AMPs) as a potential alternative to conventional antibiotics.^3,4^ These AMPs are small, typically cationic proteins that can show a broad range of activities against bacterial strains. Many AMPs primarily target the cell membrane in their mechanism of action, causing bacterial cell death through membrane permeabilization.^5,6^ However, some AMPs are believed to have intracellular targets, such as nucleic acids.^7−19^ Despite the potential therapeutic promise of AMPs, one challenge to their broader use in therapeutic applications has been the difficulty in rationally designing more active peptides in part due to the heterogeneous and amorphous nature of the bacterial cell membrane targeted by many peptides. However, AMPs with intracellular targets may provide more ready opportunities for rational design.

**Figure 1:**
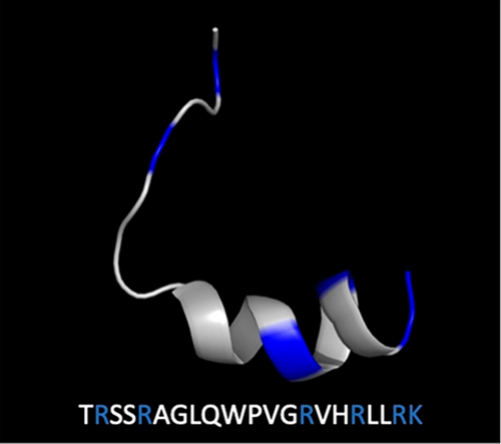
Representative structure of the BF2 peptide taken from a previous molecular dynamics simulation of the peptide interacting with a lipid membrane;^28^ positively charged residues are shown in blue.

In this work, we present a series of initial design studies focused on the AMP buforin II (BF2) (Figure 1). BF2 is one of the most thoroughly studied AMPs believed to have an intracellular target.^20^ Previous research has shown that BF2 appears to enter bacterial cells with relatively minimal membrane disruption and target intracellular nucleic acids.^21−23^ In previous work, we used a combination of molecular dynamics (MD) simulations and electrostatics calculations to characterize the interactions between BF2 and its potential nucleic acid targets,^23−25^ an approach similar to others used to analyze DNA-binding affinity for various AMPs.^13,26,27^

**Figure 2:**
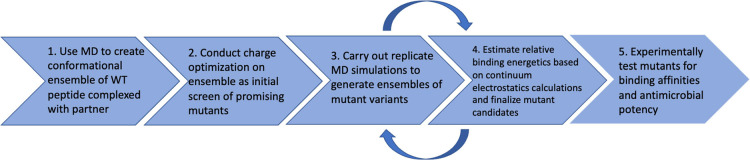
Workflow used to predict and evaluate BF2 variants with differential binding affinities to DNA.

Here, we focus on developing a computational framework to predict BF2 arginine mutations that increase BF2 affinity to nucleic acids. Our framework builds on the assumption that the affinity between BF2 and nucleic acids is governed primarily by electrostatic interactions and that the rational substitution of a neutral residue with arginine at particular positions may predictably modulate BF2/DNA electrostatic binding affinity through changes in both monopole and charge distribution. The choice to consider arginine rather than lysine substitutions to alter monopole is driven by prior work demonstrating that BF2 variants with Lys → Arg substitutions generally showed increased binding affinity.^29^ Our workflow combines MD simulations and continuum electrostatics calculations integrated with charge optimization protocols and experimental measurements (Figure 2). We implement this approach to predict and experimentally validate the binding affinities of two BF2 variants, T1R and L8R, with the ultimate goal of employing it to rationally design more active versions of AMPs with nucleic acid targets. Furthermore, by predicting BF2 mutations that lead to differing DNA affinity—with T1R predicted to improve binding robustly while L8R predicted to minimally, if at all, do so---these studies allow us to further evaluate the hypothesis that the enhanced DNA affinity of designed BF2 variants is peptide sequence-specific and not merely due to increasing the overall monopole charge on the peptide.

## Methods

As described above, Figure 2 shows the overall design workflow used in this work. For MD simulations used in steps 1 and 3, the GROMACS software package^30−33^ (versions 5.0.5, 2016.3, and 2018.3) was used. For continuum electrostatics calculations used in steps 2 and 4, a single-grid red-black successive over-relaxation finite difference solver of the linearized Poisson–Boltzmann^34^ was used (with Delphi version 8.4^35^ additionally used for validation). Throughout the process, structures were visualized using PyMol^36^ and VMD,^37^ and data analyses and plots were done using Matlab (The Mathworks, Inc., Natick, MA) and Microsoft Excel (Microsoft, Inc., Redmond, WA). Other software specific to a given step in the workflow is described below.

Here, we describe the specific methods used within each step:

### Step 1: MD Simulations To Create Initial WT BF2/DNA Ensembles

The MD simulations used to generate WT conformational ensembles were carried out as part of previously published work.^24^ To briefly summarize, the starting model for BF2 (TRSSRAGLQWPVGRVHRLLRK, with an F10W mutation included in past studies for spectroscopic experimental assays) complexed with a 21-base pair DNA duplex was obtained via homology modeling from a segment of a nucleosome core particle complexed with a fragment of DNA (PDB ID 1AOI(38)). The histidine at position 16 was modeled as the neutral delta tautomer, and all titratable groups were modeled in standard titration states at physiological pH, yielding an overall BF2 monopole of +6. After 100 steps of steepest descent minimization, three replicate simulations were carried out for 250 ns each using GROMACS^30−33^ with AMBER03 force field parameters^39^ and the TIP4P-EW water model.^40^ These simulations had a cubic box with an edge length of 8.9 nm (yielding roughly 91,000 atoms including the fourth fictitious water site), a 0.1 M NaCl concentration beyond initial charge neutralization, and an equilibrium temperature and pressure of 310 K (maintained by a velocity-rescaling thermostat^41^) and 1 atm (maintained by a Berendsen barostat), respectively. A 2 fs time step was used, with all bonds constrained via the LINCS algorithm.^42^ Cutoffs for van der Waals and for switching to long-range particle mesh Ewald electrostatics^43^ were set to 10 Å.

**Table 1:** Summary of Each Variant Simulated, Including Number and Length of Replicate Simulations. Starred Simulations Are from Prior Work^24^ and Were Used in Steps 1 and 2

BF2 variant	deprotonated H16		protonated H16	
	# replicates	time (ns)	# replicates	time (ns)
WT	4	250(3)*, 100 (1)	3	50
T1R	6	100 (4), 50(2)	5	50
A6R	3	50	3	50
L8R	4	50	5	50
Q9R	4	100	3	50

### Step 2: Charge Optimization

From each simulation in step 1, snapshots were extracted every 3.5 ns beginning at 200 ns, for a total of 15 snapshots per replicate or 45 snapshots total in the ensemble used for charge optimization. For each extracted snapshot, constrained, partial charge optimization was carried out to serve as a coarse filter to efficiently estimate the effect on BF2/DNA electrostatic binding free energy if each neutral BF2 side chain was, in turn, to bear a positive charge.

Electrostatic charge optimization^44,45^ uses a continuum electrostatics framework to determine the theoretical charge distribution on a molecule or molecular moiety that maximizes its affinity to a given binding partner. Conceptually, it optimally balances unfavorable polar desolvation costs with favorable electrostatic intermolecular interactions to yield a minimum in the electrostatic binding affinity. The theory has been comprehensively studied,^46,47^ applied,^48−54^ and reviewed^55^ elsewhere, and here we provide a very brief overview.

Using a rigid binding model within the continuum electrostatic framework, in which binding partners are represented by low-dielectric cavities with (atom-centered) embedded point charges within a high-dielectric solvent, the electrostatic binding free energy can be written as a sum of three matrix/vector products:
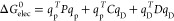
1where *q*_p_ and *q*_D_ are vectors containing the peptide and DNA point charges, respectively, and *P*, *C*, and *D* are the unit potential difference matrices for the peptide desolvation, solvent-screened complex interaction, and DNA desolvation upon binding, respectively. In this work, we wished to optimize only the atoms within a single peptide side chain at a time, leaving all other peptide charges and DNA charges fixed. In such a case, *q*_p_ can be partitioned into a variable portion, *q*_s_, and a fixed portion, *q*_t_, and eq 1 can be equivalently expressed as follows.
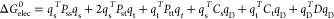
2where *P*_ss_, *P*_st_, and *P*_tt_ are the corresponding submatrices of *P,* and *C*_s_ and *C*_t_ are the corresponding submatrices of *C*. Matrix elements shown in eq 2 are computed by numerically solving the linearized Poisson–Boltzmann equation (LPBE) as described below. Once the matrix elements have been determined, eq 2 provides an analytical expression for calculating the electrostatic binding free energy as a function of a particular side chain’s charge distribution. This expression can be minimized via standard constrained or unconstrained optimization procedures to determine optimal charges for the corresponding side chain.

Matrix elements were computed through solving the LPBE via a single-grid red-black successive over-relaxation finite difference solver.^34^ Elements for the unit potential difference matrices were computed by charging each side chain atom to +1 in turn and solving for potentials in the bound and unbound states. When charge distributions were constant (i.e., *q*_t_ or *q*_D_), potentials were premultiplied by these distributions to yield vector elements. To solve for each matrix or vector element, a 401 × 401 × 401 grid was used with a two-stage focusing procedure in which the longest dimension of the box containing the system occupied 23% and then 92% of the grid, yielding a grid spacing of ∼2.2 grids/Å at the highest focusing. This resolution has been shown in past^24^ and current work to provide reasonable estimates of ΔΔ*G*’s that agreed well with those calculated at higher resolutions and/or using the Delphi solver.^35^ Inner and outer dielectric constants were set to 4 and 80, respectively, with Bondi radii^56^ used to generate the molecular surface with a probe radius of 1.4 Å. The ionic strength was set to 0.1 M. Though this system is highly charged and therefore may require modeling nonlinear ionic strength effects for quantitative accuracy, previous work^24^ demonstrated that nonlinear effects were somewhat systematic for these binding partners in a dilute environment and they were not considered in this design workflow, in which coarse, qualitative predictions were sufficient.

Once the matrix elements were obtained for each side chain, constrained convex optimization was carried out using the CONOPT solver^57^ (version 3.15G) within the GAMS software package (version 23.9.5, GAMS Development Corporation, Fairfax, VA). In order to roughly assess the potential for binding affinity improvement upon mutation to an arginine, the optimal (minimal) BF2/DNA electrostatic binding free energy was computed upon allowing each originally neutral side chain’s charges in turn to vary but constraining the total peptide monopole to either +7 (i.e., increasing that side chain’s charge by +1) or +6 (keeping that side chain neutral). Additionally, each varied point charge was constrained to lie within a range of −1e to +1e. We then used the difference between the optimal binding free energies between the +7 and +6 theoretical variants of a given side chain
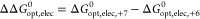
3as the metric to roughly estimate the extent to which binding affinity may improve upon a residue’s mutation to arginine. We note that this is one of many ways to estimate such a quantity, especially because this method is inherently coarse in part due to its not accounting for clear steric and conformational changes that would occur upon explicit mutation, but here, we chose to use it as our initial filter prior to more explicit models in subsequent steps.

### Step 3: Replicate MD Simulations To Generate Ensembles of Mutant Variants

Promising arginine variants of BF2 suggested by charge optimization were explicitly modeled and simulated in complex with DNA. Mutant starting structures were generated from our initial starting structure in step 1 using PyMol^36^ to create the arginine substitution. For each considered mutant, short replicate MD simulations of 50–100 ns each were carried out using the same parameters described in step 1, except the box size was roughly 9.3 nm with roughly ∼107,000 atoms including the fictitious fourth water site. A summary of all simulations is provided in Table 1.

### Step 4: Estimation of Relative Binding Energetics via Continuum Electrostatic Calculations

For each mutant and for the WT, snapshots were extracted every 500 ps from either the last 25 ns (in the case of 50 ns simulations) or the last 50 ns (in the case of 100 ns or longer simulations) of each replicate to carry forward for continuum electrostatics calculations. The BF2/DNA electrostatic binding free energy was computed for each WT and mutant snapshot via solving the LPBE using the same methods described in step 2.

### Step 5: Experimental Characterization of Peptide/DNA Binding Affinity and Antimicrobial Potency

Wild-type and variant T1R and L8R BF2 peptides selected from our computational process were then tested experimentally for their DNA binding and antimicrobial activity. For those studies, all peptides were synthesized, purified to >95% by GenScript (Piscataway, NJ), and obtained in their salt form with trifluoroacetic acid counterions. Peptide concentrations were determined using the absorbance at 280 nm.

Peptide-DNA binding was determined using a fluorescence intercalator displacement (FID) assay employed in previous studies of BF2.^23,25,29,58^ Quartz fluorescence cuvettes were rinsed with STE buffer (10 mM Tris, 50 mM NaCl, and 1 mM EDTA, pH 8.0) followed by a thiazole orange (TO) solution (0.55 μM) in STE. 2.5 mL of the 0.55 μM TO solution in STE and 5.80 μL of DNA (31.63 μM) were added to each cuvette. Fluorescence was measured using a Cary Eclipse spectrofluorimeter (excitation 509 nm; emission 527 nm) after letting the sample sit for 5 min at 25 °C. This DNA sample was titrated with 20 μL of peptide solution (82 ± 4 μM), allowing 5 min after each titration for equilibration before fluorescence readings. Titration with the peptide was continued until fluorescence had decreased to 50% of its original value. A minimum of four independent titrations were performed for each peptide. Temperature was controlled at 25 °C throughout the titration.

TOP10 **Escherichia coli** (Novagen) containing an ampicillin-resistant plasmid (pET45b) was used for both radial diffusion and microbroth dilution assays of antibacterial activity. All cultures were grown in the presence of 25 μg/mL ampicillin to minimize contamination. Bacterial cultures were grown overnight for 18–24 h in 3% w/v tryptic soy broth (TSB) in a shaking incubator at 37 °C and 150 rpm. The overnight cultures were then diluted 1:100 in 3% w/v TSB. These refresher cultures were incubated in the shaking incubator at the same settings for 2.5–3 h to reach mid log growth. The bacteria were then harvested by centrifugation at 1500 × *g* at 4 °C for 10 min and then resuspended in the buffer for the radial diffusion or microbroth dilution assays.

For radial diffusion assays, 10 mL of molten underlay agar (10 mM Na_3_PO_4_, 100 mM NaCl, 1% TSB v/v, 1% agarose w/v, pH 7.4) was mixed with 4 × 10^6^ CFU/mL of resuspended bacteria. This solution was vortexed, poured into a Petri dish, and left to solidify. Wells were then formed in this underlay using a bleach trap glass pipet, and each well was filled with 2 μL of 1 × 10^–4^ M of peptide or DI water. The Petri dishes were incubated gel side up for 3 h in a 37 °C incubator before being covered with 10 mL of molten overlay gel (2.4% w/v TSB, 1% w/v agarose). Once the overlay became solid, Petri dishes were incubated overnight (16–18 h) at 37 °C. The diameter of clearance was measured after 24 h to evaluate the relative antibacterial activity of the peptides. Data for each peptide were collected from at least two independent cultures grown on different days.

Microbroth dilution assays were performed in 96-well plates. A series of twofold peptide dilutions were performed across rows of the plates. 100 μL of liquid testing medium (LTM) and bacteria solution and 10 μL of diluted peptide were added to wells. The LTM used was STE buffer (10 mM Tris, 50 mM NaCl, 1 mM EDTA, pH 8.0) inoculated with 4 × 10^6^ CFU/mL resuspended **E. coli** bacteria. Final peptide concentrations ranged from 0.0883 to 93.6 μM. Each concentration was tested in triplicate on each plate. The plates were incubated in a nonshaking 37 °C incubator for 1 h. Then, 100 μL of fresh TSB was added to each well. The plate was incubated in the nonshaking incubator for the next 24 h. The OD_600_ of the wells after incubation was measured using a SpectraMax M3 microplate reader (Molecular Devices). A threshold OD_600_ of 0.1 was used to determine the minimal inhibitory concentrations. Data for each peptide were collected from at least three independent cultures grown on different days.

## Results

The aim of our workflow is to create BF2 variants, each with an arginine substituted for a neutral residue, with differing DNA-binding affinities, with the ultimate goals of (1) determining whether a computational workflow based on electrostatics can be effective in designing tighter DNA-binding peptides, (2) beginning to determine relationships between DNA-binding affinity and potency, and (3) testing the hypothesis that binding affinity depends on not only the overall monopole but also the specific sequence giving rise to an overall monopole. Here, we describe the results obtained from each step of the design workflow shown in Figure 2.

### Step 1: MD Simulations To Create Initial WT BF2/DNA Ensembles

**Figure 3:**
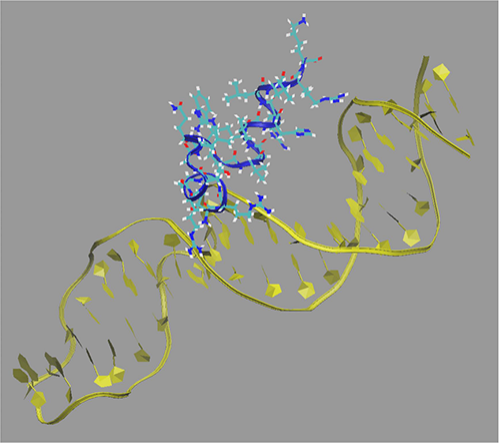
Sample snapshot (replicate 1, 200 ns) from a WT BF2/DNA complex simulation carried out as part of prior work.^24^ Peptide shown in atom colors, with the backbone highlighted in blue. DNA is shown in yellow, and water and ions have been omitted for clarity.

An initial wild-type BF2/DNA conformational ensemble used as a starting point for design predictions was generated using the equilibrated portions of three replicate 250 ns MD simulations from previously published work.^24^ The ensemble consisted of 15 snapshots sampled from the last 50 ns of each simulation for a total of 45 snapshots. Figure 3 shows a sample snapshot (200 ns) from one of the replicate simulations, with water and ions removed for clarity.

### Step 2: Charge Optimization

Constrained, partial, electrostatic charge optimization was carried out as described in the Methods section for each of the 45 snapshots within the ensemble. Each side chain with a neutral formal charge on the peptide was considered in turn, except for TRP10 and PRO11 due to the roles that these residues play in experimental quantification and peptide translocation and activity, respectively.^21,22,58^ For glycine residues, both hydrogens off the α carbon were simultaneously optimized to provide multiple feasible solutions by considering more than one atomic center. For each snapshot, a rough prediction of the effect of an arginine substitution at a given position was estimated by calculating ΔΔ*G*_opt, elec_^0^, the difference between the optimal peptide/DNA binding free energy in each conformation when the side chain’s monopole increased by +1 and the optimal binding free energy when the side chain retained its wild-type monopole as seen in eq 3 in the Methods section.

**Figure 4:**
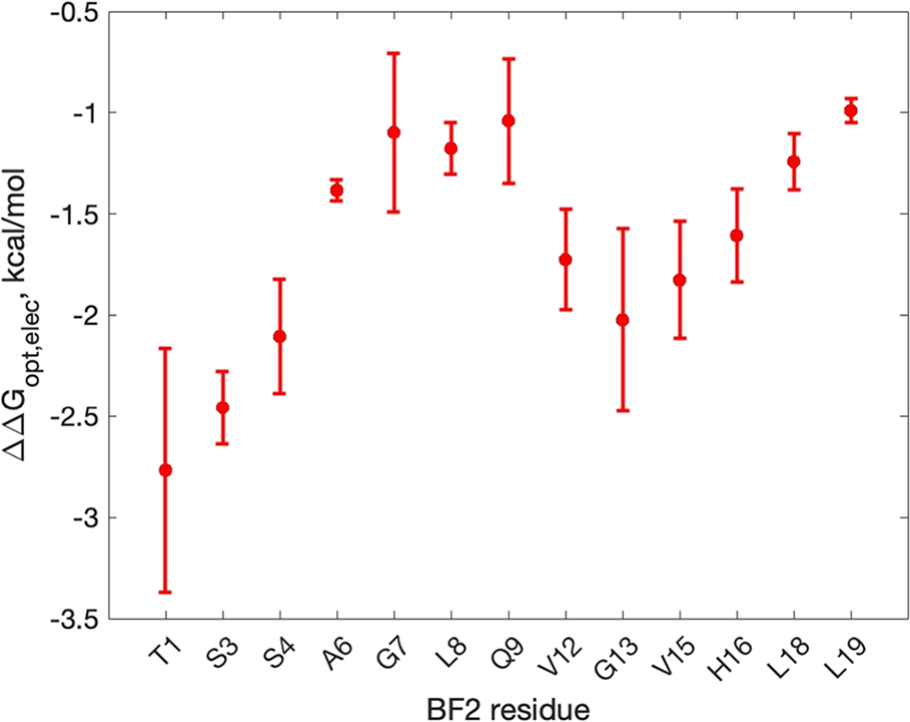
ΔΔ*G*_opt, elec_^0^, in kcal/mol for each BF2 side chain considered in electrostatic charge optimization calculations. A more negative value for ΔΔ*G*_opt, elec_^0^ indicates a greater improvement upon increasing peptide monopole by +1 in the hypothetical optimal BF2/DNA electrostatic binding free energy. Error bars show standard error over three replicates.

Figure 4 shows the average ΔΔ*G*_opt, elec_^0^ for the substitution of each residue considered across all snapshots. The positions with the most negative ΔΔ*G*_opt, elec_^0^ are predicted to improve affinity the most upon becoming positively charged, assuming both optimal interactions and retention of shape and conformation. This coarse model suggests that a single positively charged substitution at any position would increase binding affinity, which is reasonable given the substantial negative monopole of DNA. However, the extent to which the binding improves appears to be position-dependent, with positions closer to the N-terminus (e.g., THR1 and SER3) predicted to yield greater affinity gains than central positions (e.g., ALA6, GLY7, LEU8, and GLN9) or C-terminal positions (e.g., LEU18 and LEU19). This result predicts that while a peptide’s overall monopole affects DNA binding affinity, the precise distribution of charges for a given peptide monopole would significantly modulate it as well.

### Steps 3 and 4: Replicate MD Simulations To Generate Ensembles of Explicit Mutant Variants and Estimation of Relative Binding Energetics via Continuum Electrostatic Calculations

Steps 3 and 4 in the workflow were iterated until sufficiently robust mutant candidates were identified for experimental testing. In order to explicitly test whether DNA-binding affinity depended on the overall sequence monopole or more subtly on the sequence giving rise to that monopole, we wished to identify two mutants for experimental testing with differing binding affinities but with the same +7 monopole—one that was predicted to more substantially improve binding and another that was predicted to improve binding to a minimal extent. Replicate simulations of explicit mutants that appeared promising for either of these outcomes based on charge optimization were carried out, and their electrostatic binding free energies were computed for snapshots extracted every 500 ps from the last 25 or 50 ns of each replicate by solving the LPBE. Typical RMSD values in the considered timeframes calculated over protein alpha carbons and DNA phosphate atoms ranged from roughly 2–7 Å, which was roughly comparable to the range observed in WT simulations of 5–10 Å.^24^ To enable comparison with the WT binding free energy, snapshots were also extracted for computing electrostatic binding free energies from the last 50 ns of the three WT simulations used for charge optimization as described in the Methods section, and from the last 50 ns of an additional 100 ns WT simulation that was carried out subsequently to validate the robustness of the estimate of the WT binding free energy.

#### Candidate for Substantial Improvement in Binding Affinity

We first sought to predict positions that, when mutated to arginine, would substantially improve binding affinity, starting with THR1, as the charge optimization results shown in Figure 4 indicated that it had the most negative ΔΔ*G*_opt,elec_^0^. Replicate simulations (*N* = 6) of T1R BF2 were conducted, and the average resulting electrostatic binding free energy, shown in Table 2, is indeed more negative than that calculated from replicate simulations of WT BF2 (−23.5 vs −17 kcal/mol). Though this improvement is statistically significant to within *p* ≤ 0.05, the standard error for the WT binding free energies is substantial. However, previous work^25^ used similar methods to compute electrostatic binding free energies on five replicate simulations (with 11 snapshots each) of WT BF2/DNA and yielded replicate average values that were within the range found here for the WT system, providing additional evidence for the robustness of this difference. Based on these results, T1R remained a promising mutant to substantially increase affinity.

**Table 2:** Average Computed Electrostatic Binding Free Energies (kcal/mol) of BF2 Variants with DNA Using Snapshots from MD Simulations of WT or Mutant Peptide^a^

BF2 variants	with H16 neutral (kcal/mol)	with H16 protonated (kcal/mol)
**WT**	**–**17 ± 3	**–**19.6 ± 0.6
**T1R**	**–**23.5 ± 0.6	**–**24 ± 1
A6R	–23 ± 3	–24 ± 2
**L8R**	**–**18.6 ± 0.9	**–**21 ± 1
Q9R	–18 ± 2	–25 ± 2

a Uncertainty is the standard error across the corresponding number of replicates shown in Table 1. Variants selected for experimental testing are highlighted in bold.

#### Candidate for Minimal Improvement at Best in Binding Affinity

We next sought to determine a mutant whose binding affinity improvement would be minimal relative to WT. Promising candidates would be those positions with the least negative ΔΔ*G*_opt, elec_^0^ and included central and C-terminal positions as shown in Figure 4. L19R was discarded as a candidate after continuum electrostatic calculations on snapshots from an initial preliminary simulation showed a predicted electrostatic binding free energy similar to that of T1R. A6R’s predicted binding free energy (Table 2) was similar to that of T1R, also demonstrating the importance of explicit mutant simulations to follow up on the coarse charge optimization predictions. However, L8R and Q9R retained similar binding free energies relative to wild type with modest improvements on average that were not statistically significant and were pursued further as promising candidates for minimal binding improvement.

#### Considering the Effects of Histidine Protonation on Design Candidates

To increase confidence in our predictions, we carried out additional simulations and free energy calculations for WT and candidate mutant (T1R, Q9R, and L8R) peptide/DNA complexes in which we protonated the histidine at position 16. Although previous work on BF2^24^ has assumed that histidine to be in a neutral state, binding to polyanionic DNA could shift its protonation state, which could impact our binding predictions. The effects of HIS16 protonation on A6R were also considered to see if it remained similar to that of T1R across HIS16 protonation states. These results are also shown in Table 2.

Despite some shift in the predicted absolute binding affinity of WT BF2 upon histidine protonation, enhanced binding of T1R is predicted regardless of histidine protonation state (significant to *p* ≤ 0.05 for the protonated case as well). A6R showed similar binding affinities to T1R across both protonation states but with greater variability, and therefore, it was not chosen for experimental testing. For the candidates for minimal improvement, only L8R showed consistently modest binding enhancement at best, regardless of the potential histidine protonation state. In particular, the effect of Q9R mutations appeared to be especially sensitive to the histidine charge. Therefore, L8R was put forward as the candidate mutant predicted to yield minimal improvement in DNA binding relative to WT for experimental validation in this work. Interestingly, the effect of histidine protonation on DNA binding was not systematic. For example, protonating HIS16 appeared to have no effect on the binding of T1R BF2, but it was predicted to greatly improve the binding of Q9R BF2. This suggests that there may be some residue-coupled effects on DNA binding. We explore possible structural explanations for this coupling in the Discussion section.

In summary, our mutant candidates put forth for experimental testing were T1R and L8R, with the order of predicted DNA binding affinities ranging from T1R (strongest), L8R,WT (weakest).

### Step 5: Experimental Characterization of Peptide/DNA Binding Affinity and Antimicrobial Potency

**Figure 5:**
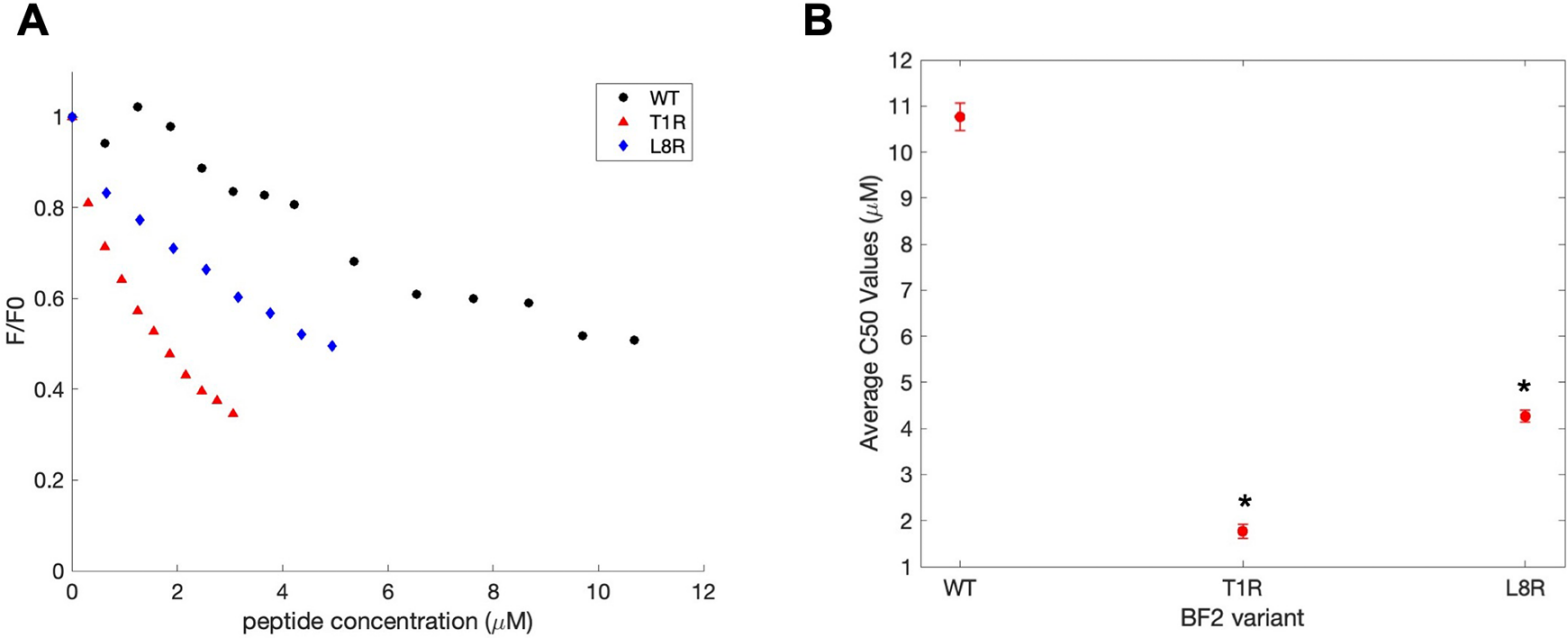
(A) Representative data from FID peptide-DNA binding experiments, showing changes in relative fluorescence (*F*/*F*_0_) with increasing peptide concentration. (B) Average *C*_50_ of T1R and L8R BF2 variants and WT BF2 in FID assay. *C*_50_ was calculated by averaging the peptide concentrations at which initial fluorescence was halved (*n* ≥ 4 for each peptide). Error bars represent the standard error. Asterisks indicate a significant difference from WT (*p* ≤ 0.05).

In order to evaluate our computational predictions, we used a fluorescent intercalator displacement (FID) assay to experimentally compare the DNA binding of WT, T1R, and L8R BF2. This approach has been previously used to characterize the DNA binding of BF2 variants.^23,25,29,58^ In these experiments, we measured the ability of each peptide to displace the fluorescent intercalator thiazole orange (TO) from DNA. The C_50_, or the average concentration of peptide needed to reduce TO fluorescence by half, is given for each BF2 variant in Figure 5; a decreased *C*_50_ implies stronger peptide-DNA interactions. Both T1R and L8R show significantly increased DNA binding (*p* ≤ 0.05) relative to wild-type BF2, though the L8R increase is indeed more modest, in line with the predictions; T1R also showed significantly increased binding compared to L8R (*p* ≤ 0.05).

While FID measurements confirmed the ability of our approach to successfully design BF2 variants with different increased DNA binding affinities, our long-term interest is to develop AMPs with enhanced antimicrobial activity. To the best of our knowledge, previous work has not attempted to engineer more active AMPs by increasing their nucleic acid interactions. However, BF2 mutations with decreased DNA binding typically show decreased activity.^23^ We have also noted that the overall activity of BF2 variants is based on a balance of their membrane translocation, membrane permeabilization, and DNA binding.^58^

**Figure 6:**
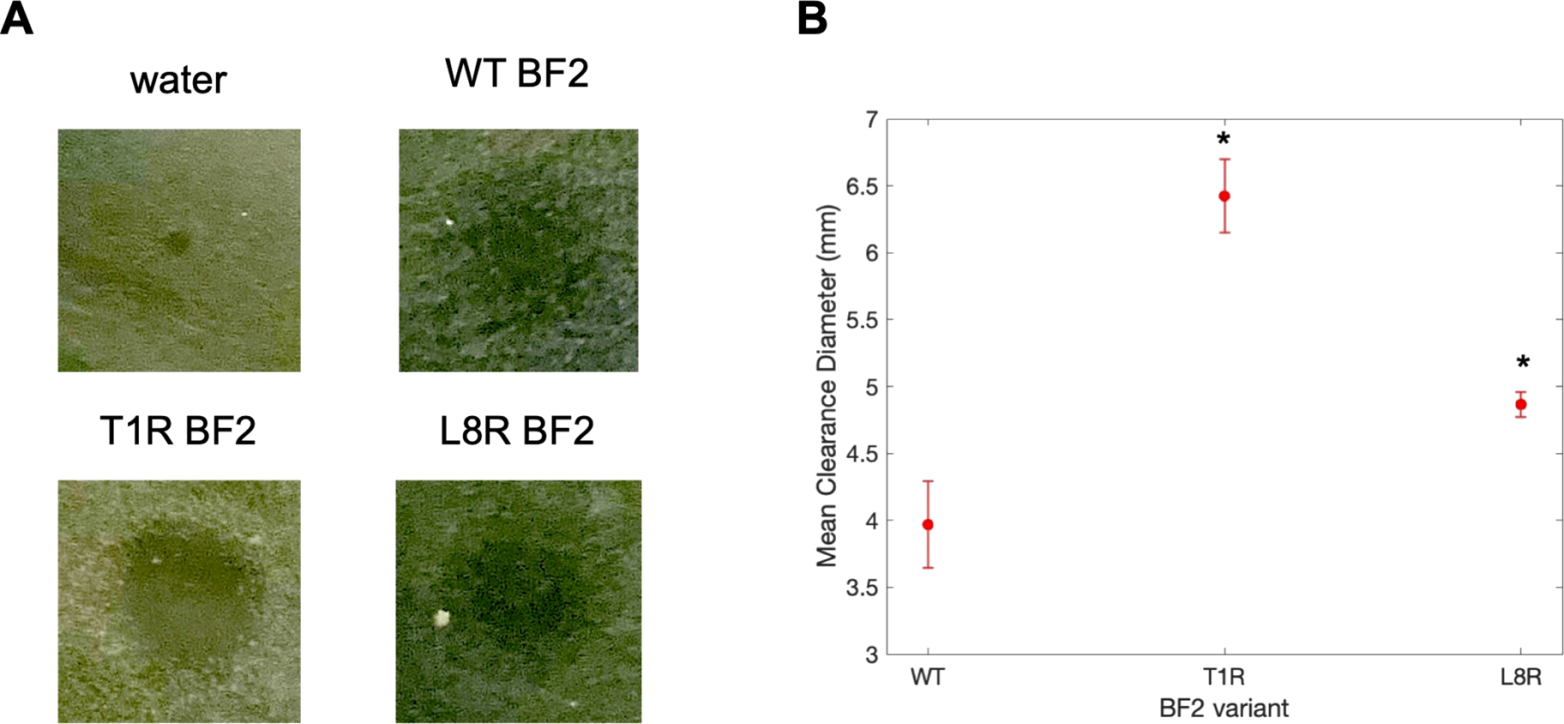
(A) Images of representative wells from a radial diffusion assay plate containing water and WT BF2 and T1R and L8R BF2 variants. Note that contrast and brightness were altered in identical ways for all four well images to improve image quality. (B) Mean clearance diameter in mm of T1R and L8R BF2 variants and WT BF2 in RDA. Mean clearance diameter was calculated by averaging the clearance diameters of each sample (*n* = 6 for each peptide). The error bars represent the standard error. Asterisks indicate a significant difference from WT (*p* ≤ 0.05).

**Figure 7:**
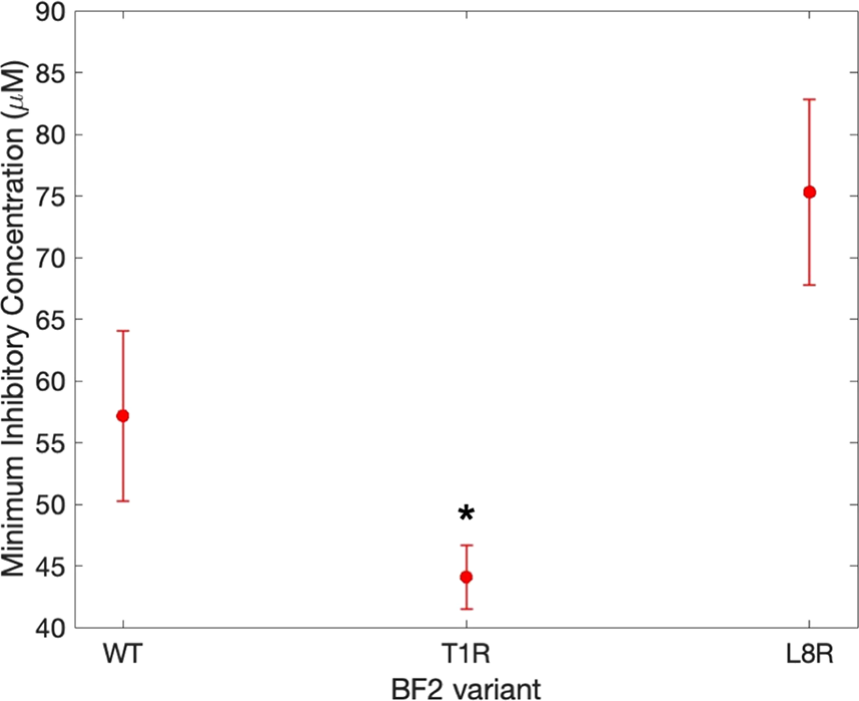
Minimum inhibitory concentrations of T1R and L8R BF2 variants and WT BF2 in microbroth dilution assay (*n* = 3 for each peptide). Threshold concentrations were determined over nine replicates performed on three independent cultures. The error bars represent the standard error. Asterisks indicate a significant difference from WT (*p* ≤ 0.05).

Thus, we also compared the activity of WT, T1R, and L8R peptides against **E. coli** using radial diffusion (Figure 6) and microbroth dilution (Figure 7) assays. In both of these experiments, the T1R mutation led to a clear increase in activity relative to that of the WT peptide, showing an increased radius of clearance in radial diffusion measurements (Figure 6) and a decreased minimum inhibitory concentration (Figure 7). Interestingly, the results for the L8R mutation showed some difference between these two methods as it showed increased activity relative to WT in the radial diffusion assay but no significant change in activity in the microbroth assays. This variation could be due to inherent differences in the setup of these experiments as a radial diffusion experiment can be impacted by unexpected differences in the diffusion rates of peptides through solid media. In fact, as noted in the Discussion section, it is feasible that the L8R peptide could show equivalent or even decreased activity relative to wild type, as observed in our microbroth assays, despite its increased nucleic acid affinity; such a phenomenon may be due to potential changes in its other properties.

### Structural Analyses of Variant Peptides from Simulations

**Figure 8:**
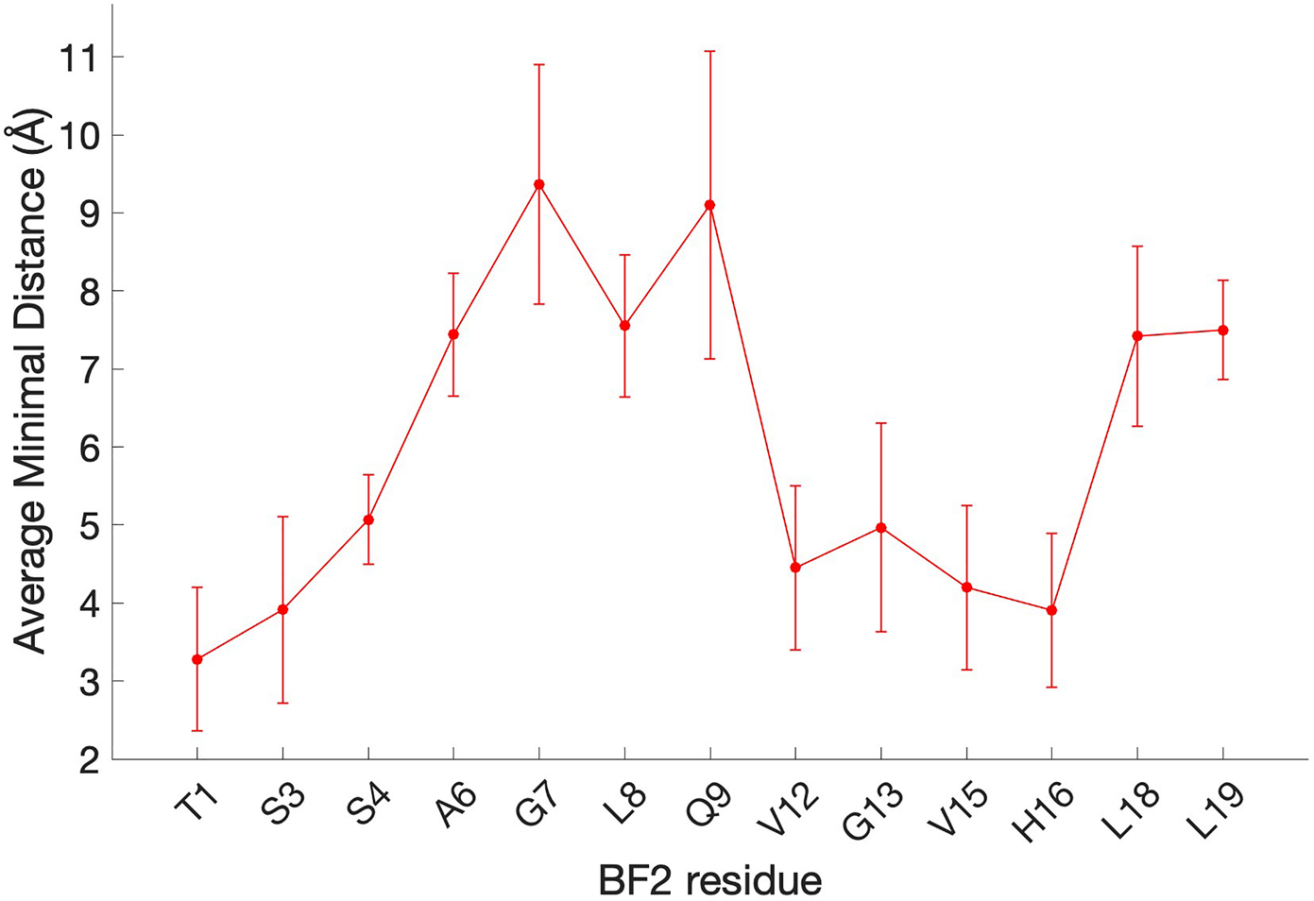
Average minimum distance (Å) between each considered side chain and DNA for snapshots extracted every 3.5 ns over the last 50 ns of each WT simulation. Error bars show standard error over the three replicates considered for charge optimization.

Our experimental results indicate that the BF2 T1R mutant had greater improvement in DNA binding affinity relative to WT than BF2 L8R, in line with our computational predictions. To better understand this observation, we carried out additional analyses on the simulations used in the prediction process. First, Figure 8 shows the average minimum distance between each side chain and DNA in the original WT simulations used to generate the ensemble for charge optimization. As the figure shows, the THR1 side chain is closer to the DNA on average than L8, indicating a greater opportunity for short-range electrostatic interactions. Interestingly, the qualitative visual pattern seen in Figure 8 is similar to that of Figure 4, in that the side chains that are closer on average also tend to have more negative predicted ΔΔ*G*_opt, elec_^0^, although the correlation is not perfect. This similarity suggests that while it may not replace the utility of charge optimization, distance analyses may be a complementary “filter” in the design workflow to highlight potentially promising candidates for mutation.

**Figure 9:**
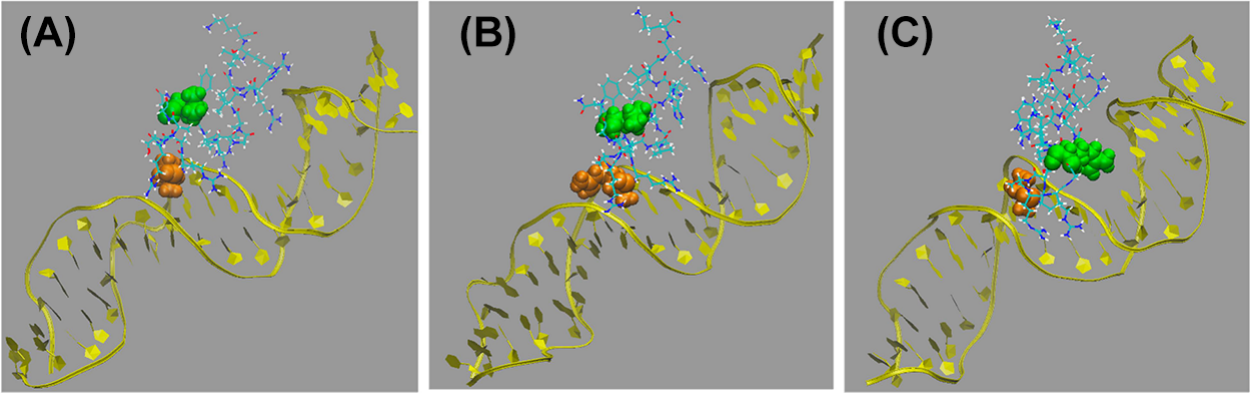
Final extracted snapshot from the first replicate of each of (A) WT (from prior work),^24^ (B) T1R, and (C) L8R variants. BF2 is shown in atom coloring, and DNA is shown in yellow. Residues 1 and 8 are colored orange and green, respectively, in each case. Water and ions have been omitted for clarity.

Figure 9 shows the final snapshot extracted for computed binding free energies (step 4) from a replicate of the WT, T1R, and L8R simulations. In each case, residues 1 and 8 are highlighted. Interestingly, in observed snapshots of nearly all simulations performed, residue 1 is generally localized within the DNA minor groove, enabling it to interact electrostatically with both flanking phosphate backbones. This observation might help to explain why increasing the monopole of this residue—already at +1 due to its being at the N-terminus—to +2 may provide favorable interactions since it is “sandwiched” between charges in the minor groove that compensate for lost solvation. Residue 8, on the other hand, remains relatively far from the DNA. As seen in Figure 9c, even though that residue approaches the DNA in the L8R simulation, it appears more water exposed and further from the phosphate backbones in the major groove. These visual data provided from MD simulations coupled with the distance analyses provide additional insight into why the T1R mutation yields greater improvement in binding than the L8R mutant.

**Figure 10:**
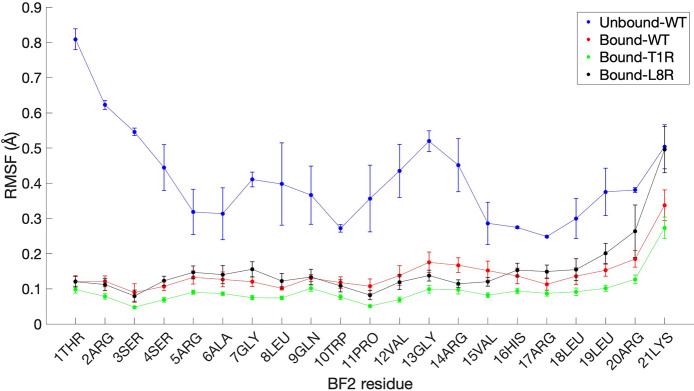
Average root-mean-square-fluctuation (RMSF), in Å, of the α carbon of each residue of simulated DNA-bound BF2 variants that were experimentally tested and unbound WT BF2. Values were calculated using the initial minimized structure for each replicate via sampling every 500 ps for the last 50 ns (in the case of simulations lasting 100 ns or more) or 25 ns (in the case of 50 ns simulations) of each replicate, and error bars show standard error over the number of replicates done (Table 1).

To better understand peptide conformational flexibility for each variant as it binds to DNA, we carried out alpha-carbon root-mean-square-fluctuation (RMSF) analyses for all replicate simulations of WT, T1R, and L8R. As a control, we also show RMSF analyses for two free, unbound BF2 simulations carried out and described as part of previous work.^24^ The results of these analyses are shown in Figure 10. Interestingly, although the N-terminal region of the unbound peptide showed the greatest movement, the N-terminus of all bound peptides was the *least* mobile region. Thus, binding to the DNA appears to “lock” the N-terminus in place perhaps due to the minor groove interactions noted above. This effect is most pronounced for the T1R mutant, suggesting a more rigid conformation and perhaps indicative of tighter interactions.

**Figure 11:**
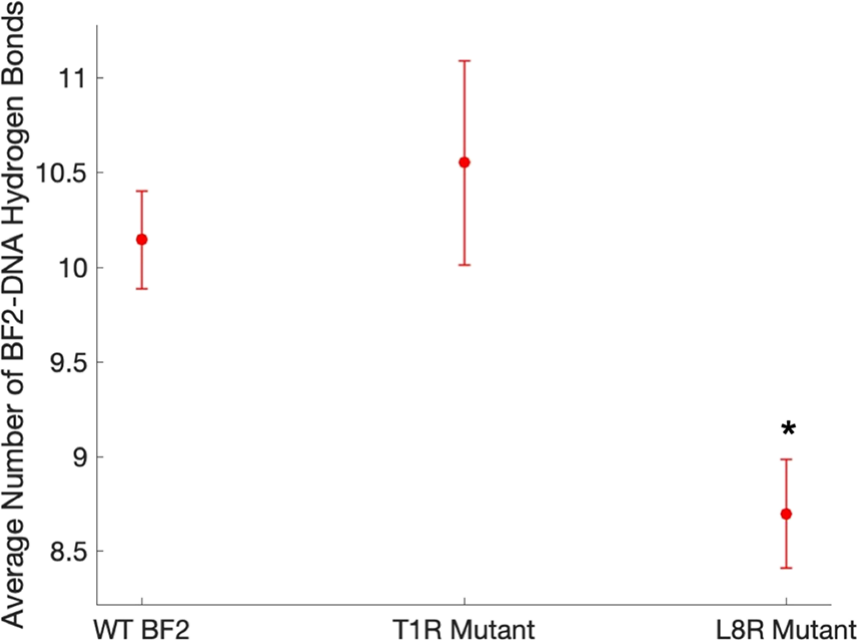
Average number of BF2–DNA hydrogen bonds across simulations of experimentally tested BF2 variants. Values were calculated every 500 ps for the last 50 ns (in the case of simulations lasting 100 ns or more) or 25 ns (in the case of 50 ns simulations) of each replicate, and error bars show the standard error over the number of replicates done (see Table 1). Nitrogen atoms were not considered acceptors in this analysis. Asterisks indicate a significant difference from WT (*p* ≤ 0.05).

Finally, Figure 11 shows the average number of peptide/DNA hydrogen bonds made for the considered portions of all WT and variant simulations. There appears to be no significant difference in the number of hydrogen bonds between the WT and T1R mutants even though the T1R mutant was experimentally shown to bind with greater affinity to DNA. Additionally, the L8R variant actually makes fewer hydrogen bonds with DNA on average than the WT (*p* ≤ 0.05) though it binds with slightly higher affinity. These data support the idea that predictions based solely on hydrogen bond quantification may not always correlate with affinity in this system and highlight potential value in our prediction workflow, which accounts more thoroughly for both short- and longer-range polar and electrostatic interactions.

## Discussion

This work provides an initial demonstration of an approach to use MD sampling with electrostatics calculations to design AMPs with an increased DNA-binding affinity. Additionally, we showed that DNA-binding affinity is dependent not only on the overall monopole of the peptide but also on the precise location of positively charged residues. Antimicrobial measurements also implied that this could be an effective approach toward designing more active AMPs, and we plan future work to better understand the relationship between peptide charge distribution, DNA-binding affinity, and antimicrobial potency for variants of BF2 and other DNA-binding peptides.

Here, we focused solely on the electrostatic component of binding when predicting mutant candidates using the hypothesis that a simplified model would suffice in a system that is clearly dominated by polar solvation and charge–charge interactions. Future work could consider the best way to account for other, nonpolar components of the binding free energy to provide more holistic insights into binding determinants. Similarly, future studies could use a broader set of starting structures beyond the homology-model-based BF2/DNA structure employed here to provide a more robust sampling of the design conformational landscape. That said, although accounting for other components of binding free energy and broader conformational sampling may be useful, particularly for more quantitative predictions, the current work demonstrates that focusing on the electrostatic components of energies with the type of ensembles collected here can be successful for design purposes in this system.

In particular, our approach was successful in predicting BF2 variants with different levels of DNA affinity. Moreover, the different affinities of the T1R and L8R variants emphasize that BF2−nucleic acid binding is peptide sequence-specific and not solely driven by the overall positive charge. A similar sequence specificity also was observed in BF2 mutants designed to have *decreased* DNA binding upon monopole decreases from single-site arginine or lysine to alanine mutations in previous work.^23^ We also found that our arginine mutation identified for the maximum increase in DNA binding (T1R) did have clearly enhanced activity in two different antimicrobial assays, giving us confidence in its increased potency. The L8R mutation with more moderate enhanced DNA binding had inconsistent results between the two activity assays that may have stemmed from different assumptions embedded in those methods related to the relative diffusion of peptides through solid agar or bacterial exposure to peptides in phosphate buffer. Regardless, we would not expect a perfect correlation between DNA binding and antimicrobial activity since our past work showed that the activity of BF2 variants was related to their membrane translocation and permeabilization in addition to their DNA binding.^58^ To this end, we are working toward integrating predicted membrane interactions into our AMP design strategy. Nonetheless, the activity of the T1R peptide provides evidence that an AMP design approach can produce promising candidates by focusing on one of these characteristics.

Our computational analyses suggested that the effect of histidine (HIS16) protonation on the binding free energies of arginine mutants was not systematic. For example, HIS16 protonation was predicted to improve the binding affinity far more for the Q9R mutant than for the T1R or L8R mutant. This observation suggests that the protonation of HIS16 may alter the manner in which other peptide residues interact with the DNA in a sequence-dependent manner. Indeed, preliminary distance and energetic component analyses suggest that in our simulations with protonated HIS16, the distance and binding free energy between the arginine at position 20 (ARG20) and the DNA are decreased more for the Q9R variant than for the T1R variant (data not shown). In other words, protonating H16 indirectly affects the contributions of a different residue, ARG20, in a mutant-specific manner. Future experimental work can measure the extent of histidine protonation in various complexes, and ongoing computational work can continue to understand and incorporate the effects of the histidine titration state on conformational sampling into the design framework. Additionally, H16F variants can be designed^59^ and experimentally tested as controls that are not titratable at this position.

In conclusion, this work has demonstrated a design workflow that combines MD simulations, charge optimization, continuum electrostatics, and experimental measurements to create mutants of a membrane translocating AMP with altered DNA-binding affinity and potency. In ongoing work, we aim to ultimately build on this approach to generate additional mutants of BF2 and other DNA-binding AMPs to better understand the relationship between DNA binding and antimicrobial potency. We recognize the relatively high MIC values for the parent BF2 peptide and our designed variants, at least under the conditions used for the antibacterial activity measurements presented here. However, the design approach utilized in this study would likely be equally effective in designing more potent versions of other DNA-binding AMPs with higher basal activity. Moreover, our proposed design framework is not specific to DNA and can therefore be applied to any peptide/target pair. Indeed, while the translocation mechanism is not fully characterized, membrane translocating peptides also must interact with the cell membrane prior to translocation. To that end, this design framework could also be adapted to generate mutant peptides to better understand the relationship between the peptide-membrane affinity and antimicrobial potency. Taken together, a holistic design of AMP-membrane and AMP-DNA interactions that provide maximal potency may serve as a useful strategy to engineer AMPs with optimal potency.
